# Sero-molecular epidemiology of hepatitis E virus in pigs and human contacts in Ghana

**DOI:** 10.1186/s42522-021-00043-w

**Published:** 2021-06-22

**Authors:** Richmond Yeboah, Augustina Angelina Sylverken, Michael Owusu, Philip El-Duah, Vitus Burimuah, Yaw Frimpong, Jones Lamptey, Isabella Eckerle, Benjamin Meyer, Christopher Antwi, Olivia Agbenyaga, Raphael Folitse, Benjamin Emikpe, Samuel Kingsley Oppong, Yaw Adu-Sarkodie, Christian Drosten

**Affiliations:** 1 Kumasi Centre for Collaborative Research in Tropical Medicine, Kwame Nkrumah University of Science and Technology, Kumasi, Ghana; 2 Department of Theoretical and Applied Biology, Kwame Nkrumah University of Science and Technology, Kumasi, Ghana; 3 Department of Medical Diagnostics, Kwame Nkrumah University of Science and Technology, Kumasi, Ghana; 4 Institute of Virology, Charite, Berlin, Germany; 5 School of Veterinary Medicine, Kwame Nkrumah University of Science and Technology, Kumasi, Ghana; 6 Geneva Centre for Emerging Viral Diseases, Geneva, Switzerland; 7 Institute of Virology, University of Bonn Medical Centre, Bonn, Germany; 8 Department of Animal Science, Kwame Nkrumah University of Science and Technology, Kumasi, Ghana; 9 Department of Agroforestry, Kwame Nkrumah University of Science and Technology, Kumasi, Ghana; 10 Department of Pathobiology, School of Veterinary Medicine, Kwame Nkrumah University of Science and Technology, Kumasi, Ghana; 11 Department of Wildlife and Range Management, Kwame Nkrumah University of Science and Technology, Kumasi, Ghana; 12 Department of Clinical Microbiology, Kwame Nkrumah University of Science and Technology, Kumasi, Ghana

**Keywords:** One health, Zoonoses, Livestock, Infectious disease reservoirs, Viral hepatitis

## Abstract

**Background:**

Hepatitis E virus (HEV) is among the leading causes of viral hepatitis in most developing countries. Zoonotic acquisition of HEV genotype 3 from swine has come into focus more recently. Available studies on HEV in Ghana and other countries in the region do not provide enough information towards understanding the epidemiology of HEV in human and animal populations. Towards this end, we conducted a comparative cross-sectional study to determine the seroprevalence and risk factors associated with HEV exposure, both in swine and humans working on pig farms in typical local settings. The presence of viral RNA in human and swine samples was also evaluated, along with classification of viral sequences from HEV-positive samples.

**Methods:**

Structured questionnaires soliciting information on pigs reared, as well as socio-demographic information including age, sex and educational background of humans was collected. A total of 10 ml and 5 ml of whole blood was collected from pigs and human participants respectively. ELISA and real-time RT-PCR were performed on the sera for the qualitative detection of IgG antibodies to hepatitis E virus and viral RNA, respectively.

**Results:**

Five hundred and forty-four (544) human participants including 264 swine contacts and 280 swine non-contacts were enrolled in the study. Although the proportion of HEV IgG antibodies was higher in contact groups (114; 54.3%) than non-contact groups (96; 45.7%), a multivariate analysis did not show any significant difference. No HEV RNA was detected in human samples. Similarly, 720 pigs were sampled from 18 farms located in five regions in Ghana. Twenty-three (23) of the pigs (3.2, 95%CI = 2.0–4.8) were positive for HEV RNA by real-time RT-PCR testing. Sequences obtained from HEV-positive samples were found to share high sequence identities with each other and clustered with other genotype 3 viruses indicating the existence of circulating zoonotic genotype 3 viruses on farms. Although we did not find evidence of pig to human transmission of HEV genotype 3, the presence of this genotype in pigs shows the potential for possible zoonotic transmission in African farm settings and buttresses the importance of active surveillance for the infection among at risk populations.

## Background

Hepatitis E virus (HEV) is the leading cause of viral hepatitis in much of the developing world [1]. Even though the infection has a worldwide distribution, the highest prevalence rates are observed in developing countries particularly in East and South Asia and Africa [2–7]. Annually, an estimated 20 million HEV infections occur worldwide leading to approximately 56,600 deaths [8]. HEV infection is usually self-limiting, but may develop into fulminant hepatitis with case-fatality rate rising from 2% in the general population to 40% among pregnant women [9]. The virus has at least 4 different types: genotypes 1, 2, 3 and 4. Genotypes 1 and 2 have been found only in humans [10]. Genotypes 3 and 4 circulate in several animals (including pigs, wild boars, and deer) without causing overt disease, and occasionally infect humans [11]. HEV is spread mainly by the faecal-oral route via contaminated water [12] and ingestion of undercooked meat or meat products derived from infected animals (e.g. pork liver) [13]. Of note, the zoonotic nature of HEV genotype 3 acquired from swine has been grossly underestimated in Europe. Not only does the virus cause persistent infection with a risk to develop liver cirrhosis in immunosuppressed patients [11]; recent studies and growing clinical experience show that the virus not only causes hepatitis, but an array of mainly peripheral neurological syndromes [14, 15].

Pigs are the most important reservoir for HEV genotype 3 [16]. However, in pigs, HEV in most cases causes only subclinical infection [17, 18]. This notwithstanding, the risk of zoonotic transmission to humans is an important public health concern [19] and warrants a search for risk factors of infection.

Better appreciation of genotype 3 during the last 10–15 years has triggered re-adjustments of serological test antigens and PCR primers [20]. Only with these improvements has it become clear that HEV prevalence in European populations is high in the range of 5% or even above [21–30]. There have been studies on HEV in Ghana [31–40], but these have not targeted genotype 3 specifically. Studies focusing on zoonotic hepatitis E in sub-Saharan Africa should therefore aim to detect genotype 3 and investigate sources of infection (swine) along with humans. Because important information on the influence of animal contact on infection cannot easily be derived from mere prevalence data, studies should try to link infections via sequencing and ask for rates and conditions of animal contact. As the geographic diversity in African countries is huge, they should cover a larger geographic space as well as places of different levels of infrastructure and development.

In the present study we employed both serological and molecular techniques to assess the prevalence of HEV in humans and swine, investigate HEV genotypes and determine risk of infection associated with working or living in close proximity to pig farms.

## Study methods

### Study areas, design, and sampling strategy

**Fig. 1:**
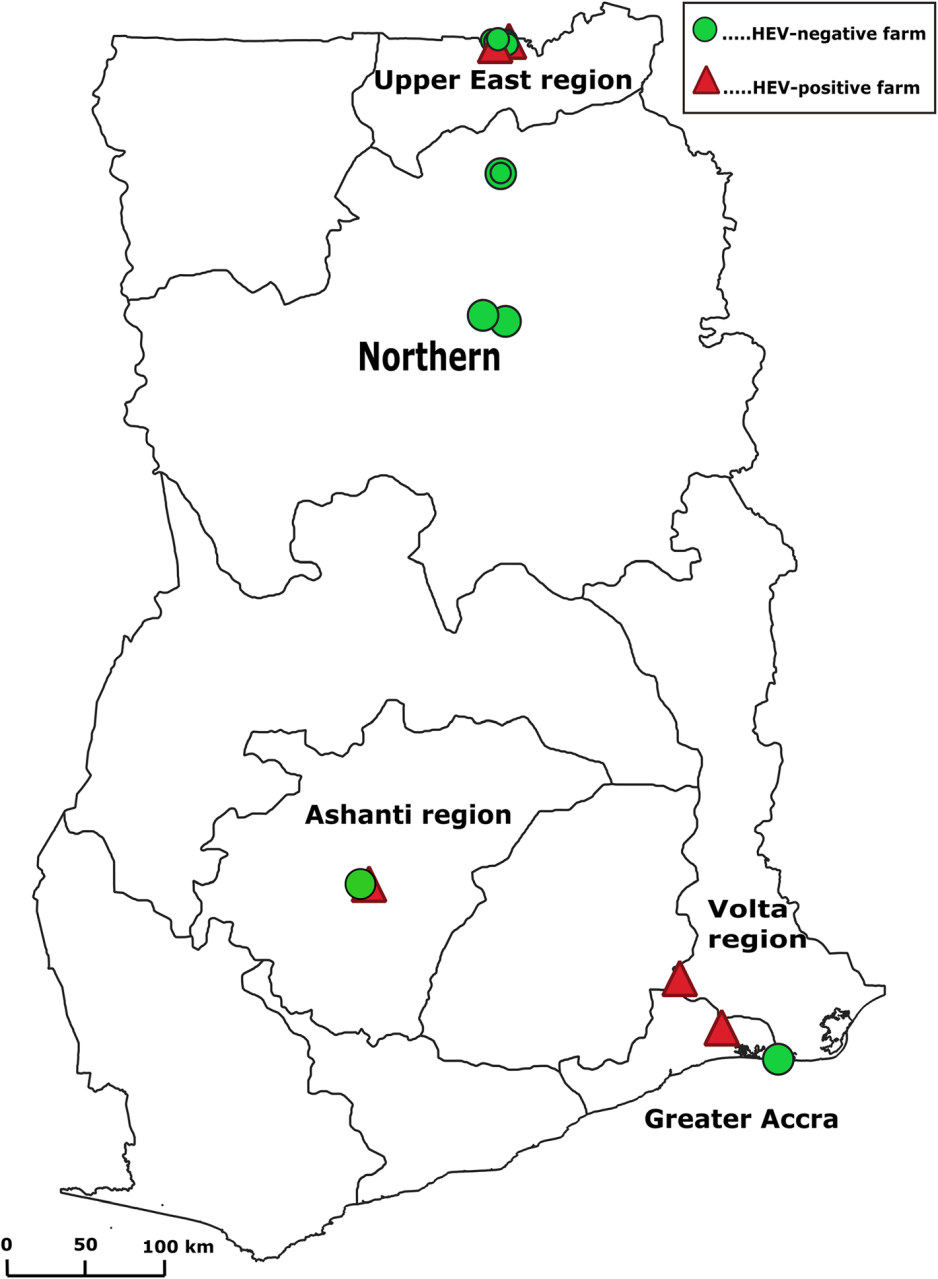
Map of Ghana showing the regions and farms where samples were collected

The research was a comparative cross-sectional study that was conducted as part of a prospective investigation of possible zoonotic agents in livestock and wildlife in Ghana. The study was conducted between June, 2015 and May, 2016 in communities having pig farms in five Regions in Ghana. The Regions were the Ashanti, Greater Accra, Northern, Upper East and Volta Region (Fig. 1).

### Study design for pig sampling

Prior to commencement of the study, a sample size of at least 124 pigs was calculated using an expected blood HEV RNA of 2.1% [41] and a confidence level of 95%. The formula used for detecting at least one positive animal was computed from Annon and Roe [42].

$$ n=\left\{1\left(1-p\right)\Big\{1{\left(1-p\right)}^{\frac{1}{d}}\right\}x\ \left\{N-\frac{d}{2}\right\}+10=120 $$

Where n is the required samples size; N is the average herd size; d is the expected number of infected animals and p is the confidence level of 95%.

The farms were selected using a simple two-stage cluster sampling technique. At the first level of sampling, Regional Veterinary Officers at all the ten regions of Ghana were contacted for information on swine populations in their respective regions. A comprehensive list of farms with swine populations was obtained and regions having farms with cumulative herd size of ≥50 pigs were selected. Each of the farms selected in the region were further examined and a subset of farms were selected taking into consideration accessibility to the farms and willingness of farm managers to have their pigs sampled. For each farm we counted the number of animals and selected representative animals based on proportion based sample size. Where particular pig pens have huge numbers, we selected every second pig until a pre-determined sample size was reached.

Each of the farms selected was visited by the research team. During these visits, the reason for the study, how the study was to be conducted and information on use of data was provided to the farm managers. Farm managers were also allowed to ask questions and were further encouraged to seek clarification on issues they were concerned about. The farms of managers who consented to the study were enrolled and every individual pig was assessed and sampled. In order to minimize possible injury to the animals, piglets less than 2 months old and heavily pregnant sows were excluded from the study. Variables assessed on pigs included number of pigs reared by the respondents, estimated age, gender and body scoring. Veterinarians assisted in measuring clinical parameters such as presence of fever, neurological disorders, diarrhoea, icterus and other signs of disease. Fever status was assessed based on rectal temperatures above 40 °C degree Celsius [43].

### Study design for human sampling

A sample size of 86 each for contact and non-contact groups was determined using an expected seroprevalence of 15% among contact groups and 2% among non-contact groups [44], a study power of 80 and confidence level of 95%. For the purpose of this study, we defined “contacts” as individuals who directly take care of the pigs and those who live together with pigs in their households. “Non-contacts” were those living in the same community/vicinity with the contact groups but not involved in taking care of pigs or living together with the pigs in their household. All contacts and non-contacts were approached and those who consented were enrolled and sampled. All respondents were interviewed with a questionnaire which had been pre-tested and revised. Variables collected from human contacts and non-contacts included age, gender, religion, occupation, highest level of education, availability of toilet facility and consumption of pork. We also asked participants about the source of their drinking water. Questionnaires were designed such that participants could select a “yes” or “No” option for each type of drinking water source.

### Laboratory investigation

A minimum of 5 and 10 ml of blood was collected from humans and pigs, respectively. All samples were transported via cold chain to the Kumasi Centre for Collaborative Research in Tropical Medicine (KCCR) where testing was carried out. Molecular analysis was performed on both pig and human samples. Additional serological analysis was performed on only human samples.

### Serological analysis of human samples

Samples were tested for the presence of IgG antibodies to HEV. The test was performed using an Enzyme-Linked Immunosorbent Assay (ELISA) for in-vitro qualitative detection of IgG antibodies to hepatitis E virus in human serum according to manufacturer’s (Axiom Diagnostics, Germany) instructions. This kit has sensitivity and specificity of about 100 and 99% respectively [45–48]. Briefly 100 μl of sample diluent was first added to micro-plates already pre-coated with recombinant antigens corresponding to the structural regions of HEV Open Reading Frame-2. Ten microliters (10 μl) of study samples including negative and positive controls were added to each pre-labelled sample wells. The plates were incubated at 37 °C and 100 μl of horse radish peroxidase (HRP) was added followed by the addition of 50 μl of chromogen A and B substrates. The final reaction was read at 450 nm. The cut off values (C.O) for the wells (samples) were calculated according to the manufacturer’s directions using the formula: C. O = Nc + 0.16 (Nc = the mean absorbance value for three negative controls).

### Molecular testing for HEV RNA in humans and pigs

Serum viral RNA was extracted from 140 μl of human and animal serum using the spin protocol of QIAamp Viral RNA Mini Kit following the manufacturer’s instructions (Qiagen, Hilden-Germany). HEV RNA detection based on a OneStep Real-Time PCR amplification was performed with reagents from Invitrogen (Thermofisher scientific, USA). The PCR testing was done by preparing a 25 μl reaction volume. The reaction volume was made up of 4.1 μl of RNase free water, 12.5 μl of 2X reaction mix, 0.4 μl of 50 mM MgSO_4_, 1.0 μl each of forward primer (5′–GGTGGTTTCTGGGGTGAC–3′) and reverse primer (5′–AGGGGTTGGTTGGRTGRA–3′), 0.5 μl of probe (5′–FAM-TGATTCTCAGCC CTTCGC–BHQ1–3′), 0.5 μl of an Invitrogen superscript III One-step RT-PCR Taq enzyme mix and 5.0 μl of the template RNA. In-house in vitro transcript of HEV RNA and RNase free water were included in each run as positive and negative controls respectively.

Master mix preparation and RNA addition were aseptically done in separate rooms to minimise chances of contamination. Cycling conditions for the reaction were; initial reverse transcription at 50 °C for 30 min, polymerase enzyme activation at 94 °C for 3 min, followed by a denaturation step at 94 °C for 15 s. Annealing was achieved at 60 °C for 30 s. The denaturation and annealing steps were repeated for 45 cycles. Acquisition was done after each annealing step. Bio-Rad CFX96 C1000 thermal cycler (Bio-Rad Laboratories Inc., Singapore) was used for the cycling and analyses of the PCR results. According to internal validation, samples were considered negative if the threshold cycle value was > 38, uncertain if between 36 and 38 and positive if less or equal to 36 [49].

### Sequencing and phylogenetic classification of derived sequences

Samples that were positive by real-time RT-PCR were further screened with a hemi-nested RT PCR assay targeting the RNA-dependent RNA polymerase (RdRp) region. This was done to generate amplicons of approximately 338 base pairs as previously described [50] for confirmation by sequencing. Amplicons were Sanger-sequenced (Microsynth Seqlab, Göttingen, Germany) and overlapping bi-directional sequences were joined and MAFFT sequence alignments done in Geneious R11 (https://www.geneious.com). Classification of derived sequences was performed by phylogenetic comparisons with reference sequences available in GenBank by Bayesian inference using the MrBayes plugin in geneiosus [51]. A general time reversible model with gamma distribution and proportion of invariable sites (GTR + G + I) was used.

### Statistical analyses

#### Swine analysis

Descriptive analysis was performed to determine the frequency and percentages of categorical variables. HEV RNA prevalence with its 95% confidence interval was computed at the animal level and farm based on a binomial distribution assumption. The association between HEV prevalence and sociodemographic or other clinical variables were tested using Fisher’s exact test or Chi-square test where appropriate. Variables which were significant were entered into a logistic regression model to determine the independent risk factors associated with HEV positivity in the pigs. Estimates from the model were expressed as odds ratio (OR) and Wald’s 95% confidence interval (CI). All statistical tests were two-tailed and *p* ≤ 0.05 were considered statistically significant. Statistical analysis was done using R statistical software (version 3.5.1, 2018).

#### Human analysis

Categorical variables were expressed as frequencies and percentages for antibodies to HEV detected. Continuous variables were expressed as mean or medians based on the distribution of the data. The association between HEV detection and contact with pigs together with other variables was analysed using Fisher’s exact test or Chi-square test where appropriate. Variables that were significant were entered into a logistic regression model. Confounding variables were monitored by evaluating the change in the coefficient of a factor after removing another factor. Estimates were expressed as OR and 95% Wald’s CI. A statistically significant result was considered when *p*-value ≤0.05.

## Results

### Sociodemographic characteristics of study participants

Our study enrolled a total of 544 study participants. The median age of participants was 35 (IQR = 25–50) with more males (303, 56.3%) than females (235; 43.5%). Majority of the participants were from the Upper East region (313; 57.5%). Two hundred and sixty-four (264; 48.5%) were in direct contact with pigs and 280 (51.5%) were not. Most of the subjects were married (374; 69.6%) and many had no formal school education (251; 46.1%).

### Hepatitis E testing in humans

All human samples were negative for hepatitis E viral RNA. Two hundred and ten participants were positive for IgG with an overall prevalence of 38.6%. A comparison of HEV antibody positivity for other risk factor variables showed significant differences for age, educational level, religion, marital status, region of participants, the type of drinking water (pipe borne water, borehole) and contact with pigs (Table 1). The frequency of HEV antibodies in contact groups (114; 54.3%) was higher than those in the non-contact groups (96; 45.7%) and the difference was statistically significant in univariate analysis (*p* = 0.041). However, adjusting for other significant variables in a multivariate logistic regression analysis left no significant difference for HEV prevalence among contact and non-contact groups. Similarly, a higher number of christians were hepatitis E antibody positive as compared to muslims and traditionalists. Although the difference was siginificant in the univariate analysis, further adjustment with other variables at the multivariate level did not show any significance. A further analysis at the multivariate level did not show significant difference for the types of water, education, religion and marital status. The region and age group were however significant risk factors of HEV positivity (Table 2).

**Table 1:** Factors associated with HEV IgG positivity in humans

Variable	Negative (%)	Positive (%)	***P***-value
Total	334	210	
**Age group (**^**a**^**)**			< 0.001*
13–32	192 (59.1)	63 (30.3)	
33–52	87 (26.8)	77 (37)	
53–90	46 (14.2)	68 (32.7)	
**Highest educational level**			< 0.001*
No formal school education	122 (36.5)	129 (61.4)	
Tertiary	27 (8.1)	8 (3.8)	
Middle School	17 (5.1)	5 (2.4)	
Junior High School	69 (20.7)	26 (12.4)	
Senior High School	43 (12.9)	11 (5.2)	
Primary School	56 (16.8)	31 (14.8)	
**Religion**			< 0.001*
No religion	5 (1.5)	7 (3.3)	
Christian	229 (68.6)	115 (54.8)	
Muslim	73 (21.9)	48 (22.9)	
Traditional	27 (8.1)	40 (19)	
**Gender (**^**a**^**)**			0.179
Female	137 (41.3)	98 (47.6)	
Male	195 (58.7)	108 (52.4)	
**Marital status of respondent**			< 0.001*
Single	106 (31.9)	30 (14.6)	
Married	210 (63.3)	164 (80)	
Cohabiting	5 (1.5)	0 (0)	
Divorced	6 (1.8)	2 (1)	
Separated	1 (0.3)	1 (0.5)	
Widowed	4 (1.2)	8 (3.9)	
**Region**			< 0.001*
Ashanti	53 (15.9)	21 (10)	
Greater Accra	23 (6.9)	4 (1.9)	
Northern Region	29 (8.7)	33 (15.7)	
Upper East	167 (50)	146 (69.5)	
Volta Region	62 (18.6)	6 (2.9)	
**Do you use pipe as your source of water (**^**a**^**)**			0.022*
No	267 (80.2)	185 (88.1)	
Yes	66 (19.8)	25 (11.9)	
**Do you use a well as your source of water (**^**a**^**)**			0.54
No	264 (79.3)	161 (76.7)	
Yes	69 (20.7)	49 (23.3)	
**Do you use a borehole as your source of water (**^**a**^**)**			< 0.001*
No	150 (45)	62 (29.5)	
Yes	183 (55)	148 (70.5)	
**Do you have toilet facility in your home (**^**a**^**)**			0.035*
No	168 (50.6)	126 (60.3)	
Yes	164 (49.4)	83 (39.7)	
**Do you use stream as your source of water (**^**a**^**)**			0.015*
No	324 (97.3)	210 (100)	
Yes	9 (2.7)	0 (0)	
**Do you consume pork (**^**a**^**)**			
No	134 (40.2)	199 (59.8)	
Yes	81 (38.6)	129 (61.4)	0.766
**Contact with Pigs**			
No	184 (55.1)	96 (45.7)	0.041*
Yes	150 (44.9)	114 (54.3)	

*Shows signirficance for variable with *p*-value < 0.05; ^a^ denotes variables that had missing data points in them. The proportions shown are based on available data provided by respondents and not on the total positive and negative for all participants. NB: All proportions calculated are based on the complete set of column data for positive and negative

**Table 2:** Independent risk factors of HEV antibody exposure

Region	Crude OR (95%CI)	Adj. OR (95%CI)	***P*** (Wald’s test)	***P*** (LR-test)
Ashanti	Reference	Reference		< 0.001
Greater Accra	0.36 (0.1,1.33)	0.29 (0.07,1.16)	0.081	
Northern Region	3.16 (1.5,6.64)	7.86 (2.8,22.04)	< 0.001	
Upper East	2.43 (1.37,4.3)	4.03 (1.67,9.76)	0.002	
Volta Region	0.29 (0.11,0.78)	0.43 (0.14,1.31)	0.139	
**Age group**			< 0.001	
Ref: 13–32				
33–52	2.61 (1.71,3.99)	3.11 (1.97,4.93)	< 0.001	< 0.001
53–90	4.25 (2.63,6.86)	5.81 (3.4,9.91)	< 0.001	

*LR* Likelihood ratio test

### Pig characteristics

Seven hundred and twenty (720) pigs were sampled from 18 farms located in five regions. Of these, 439 (61.0%) were females. Most of the pigs sampled were adults 510 (70.8%) as compared to weaners 210 (29.2%). Based on records of rectal temperature taken, 175 (24.3%) of the pigs had fever and 545 (75.7%) had no fever.

### HEV RNA detection in pigs

Twenty-three (23) of the 720 pigs (3.2, 95%CI = 2.0–4.8) were positive for HEV RNA by real-time RT-PCR. The highest number of HEV was detected on farms where most weaners were sampled. The detection rate in weaners (19/210, 9, 95%CI = 5.5–13.8%) was about ten times that in adult animals (4/510, 0.8, 95%CI = 0.2–2.0), which was significantly different (*p* <  0.001). Detection rates were similar in male vs. female animals (*p* = 0.126). A multivariate logistic regression showed weaners have 11.57 (95% CI = 3.83,34.94) odds of exposure to HEV compared to adults and this was statistically significant (*p* <  0.001) as shown in Table 3.

**Table 3:** Association between HEV detection and pig characteristics

Variable	Hepatitis E RNA		
	Negative	positive	*p*-value
Total (*n* = 720)	697	23	
**Age of animal**			< 0.001
Adult	506 (72.6)	4 (17.4)	
Weaners	191 (27.4)	19 (82.6)	
**Sex of animal**			0.126
Female	429 (61.5)	10 (43.5)	
Male	268 (38.5)	13 (56.5)	
**Rectal temperature**			0.053
Fever	165 (23.7)	10 (43.5)	
No fever	532 (76.3)	13 (56.5)	
**Presence of ectoparasites**			0.878
No	520 (74.6)	18 (78.3)	
Yes	177 (25.4)	5 (21.7)	
**Body scoring**			0.487
Emanciated	10 (1.4)	0 (0)	
Moderately fat	39 (5.6)	0 (0)	
Normal	498 (71.4)	15 (65.2)	
Obese	5 (0.7)	0 (0)	
Thin	145 (20.8)	8 (34.8)	
**Ticks**			0.345
No	661 (94.8)	21 (91.3)	
Yes	36 (5.2)	2 (8.7)	
**Icterus**			< 0.001
No	697 (100)	23 (100)	
**Respiratory distress**			< 0.001
No	697 (100)	23 (100)	
**Neurological disorder**			< 0.001
No	697 (100)	23 (100)	
**Flea**			1
No	693 (99.4)	23 (100)	
Yes	4 (0.6)	0 (0)	

### Classification of HEV sequences

**Fig. 2:**
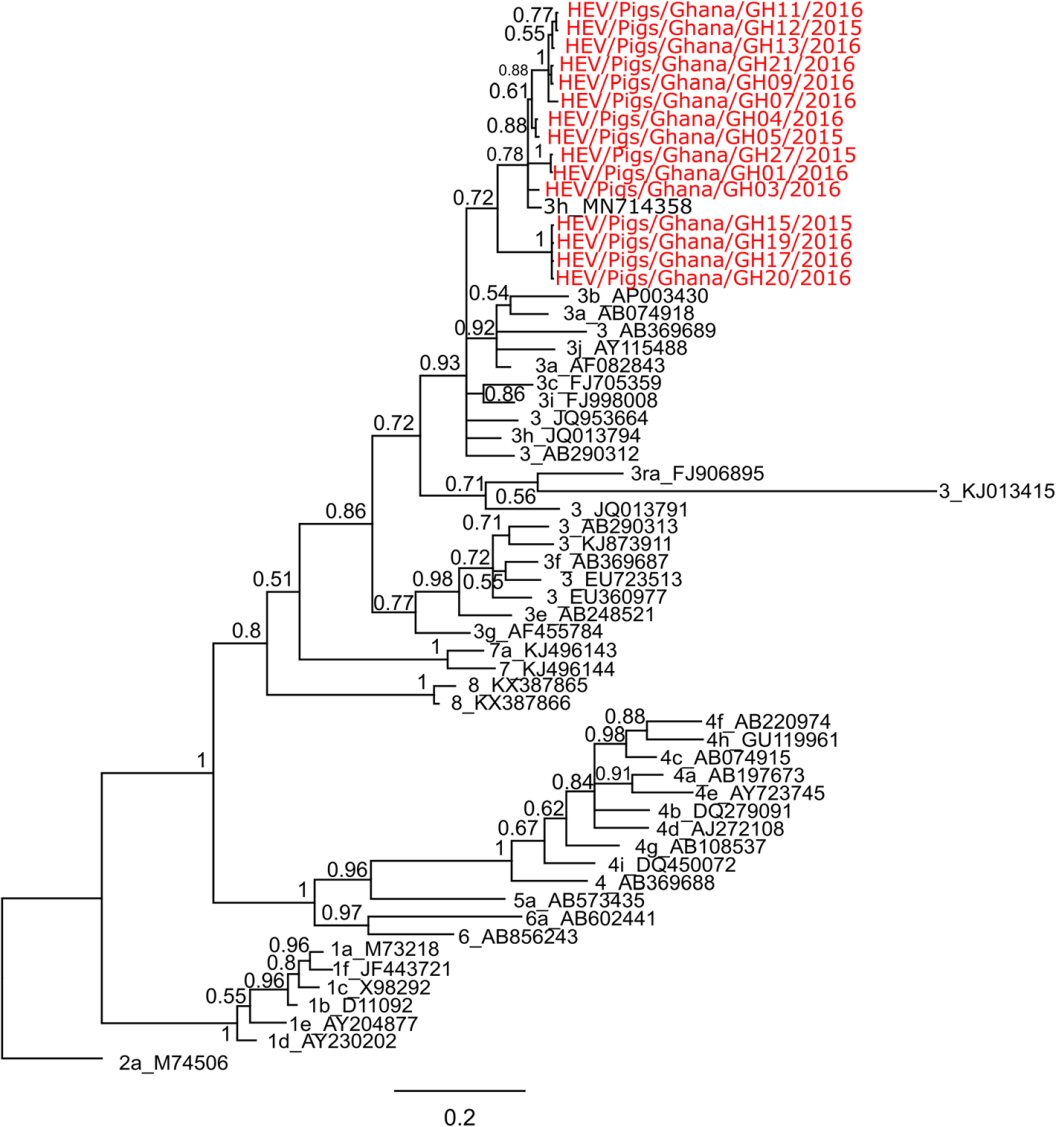
Phylogenetic comparison of sequences from this study with HEV reference sequences. The tree was generated by Bayesian inference using a general time reversible model with a gamma distribution and proportion of invariable sites (GTR + G + I). Sequences from this study are highlighted in red. Reference sequences are designated with GenBank accession numbers preceeded by genotype and subtype (when available) designation. The tree was rooted with a genotype 2 sequence

All 23 real-time RT-PCR-positive samples were also positive by hemi-nested RT PCR. The sequences obtained form this study were most similar to each other with a maximum sequence identity of 99.84% and a minimum of 87.62%. The most closely related sequence to the ones from this study was another sequence from Ghana (Accession number: MN714358) [52] with 97.43% as the highest sequence identity followed by a sequence from Germany (Accession number: FJ998008) with a sequence identity of 88.75%. The sequences from this study clustered together with other genotype 3 sequences when compared with other HEV reference sequences [53] as seen in Fig. 2. All sequences obtained in this study were submited to GenBank and assigned accession numbers MW331262-MW331276.

## Discussion

HEV is considered to be of significant public health importance in many developing countries and likely to thrive in resource-poor regions in Africa and Southeast Asia. The prevalence of anti-HEV has been extensively reviewed in Africa with rates varying from 0% in the general population [54] to 84.3% in pregnant women [55]. We recently reported a prevalence of 32.9% among jaundiced patients in Ghana [56].

Although high sero-prevalence has been reported, the source of these infections in resource-poor settings is still under investigation. Two studies conducted in the capital city of Ghana (Accra) reported total IgG prevalence of 34.8 and 38.1% among pig handlers [5, 57]. It was however not clear whether this was associated with pig farminig or common to the general population. We therefore conducted a comparative cross-sectional study to find out whether rearing or staying in close proximity to pigs is associated with exposure to HEV and to further evaluate the risk of zoonotic HEV infection in both swine and humans.

Our study identified a 3.2% prevalence of HEV RNA viremia among pig samples in five regions in Ghana with most cases from farms having a high proportion of weaners. This indicates high transmission and incidence rates during early life when maternal immunity begins to wane, which corresponds to findings from other regions with intensive hog farming [58]. Also, the prevalence of viremia was similar to studies of HEV in Europe [59]. All HEV RNA samples were identified as genotype 3, suggesting a risk of zoonotic hepatitis E like in other regions of the world [60]. Our search for swine-related factors associated with human infection therefore seems justified.

Several reports in industrialised countries support a link between presence of anti-HEV and direct contact with pigs. Studies in United States [61], Switzerland [62], Germany [63], Australia [64] found significant differences in prevalence of individuals in close contact with pigs compared to those not exposed. On the contrary other studies conducted in Sweden [44] and Brazil [65] found no significant differences between those exposed or unexposed to pigs. Data are more limited in Africa. Previous studies in Ghana and Madagascar investigated only pig contacts but no simultaneous control groups [5, 66]. One study in Uganda identified high HEV antibodies among pig contact groups as compared to non-contact groups [67].

Whereas we have not been able to detect viral RNA in any of our human subjects and thereby failed to prove transmission by sequence identity, the design of our study permits some relevant conclusions based on seroprevalence. For instance, the risk of HEV infection was independently associated with age. People aged more than 32 years had the highest exposure as compared to young groups (< 32). Knowing that antibodies against HEV persist for prolonged time [68], seroprevalence can only summarize cumulative exposures per group rather than being able to indicate recent infection [69] (it should be mentioned, however, that some authors consider anti-HEV antibodies to be short-lived and wane after ca 5 years [70]). As seroprevalence was high also in the group without direct contact, this can explain why we have not seen a correlation between direct pig contact and seropositivity. Transmission events in our study are likely to be reflected by serology on a cumulative basis, and also are assumed to include indirect exposures such as via fomites or water, as indicated by increased seroprevalence in parts of the population with lower levels of education, less favourable sanitary conditions, and no access to tap water. These are among the expected patterns of seroprevalence also for HEV genotypes 1 and 2, and these genotypes may account for a part of the seroprevalence observed in humans. Other examples such as the different seroprevalence rates between Christian and Muslim groups, however, point toward acquisition of HEV based on pork consumption. One limitation of this study was our inability to collect information on duration for contact with pigs or interaction in pig environments. Collection of this information would have improved further risk factor analysis in multivariate logistics regression. Further studies could consider inclusion of such variables in order to determine the levels of risk in contact and non-contact groups.

## Conclusion

Our data confirm previous reports of high endemicity of HEV in Ghana. Active surveillance and introduction of vaccines could be helpful to reducing active infections in human. We have also provided evidence that pigs in Ghana have infectious form of HEV circulating among them and this could pose significant risk to zoonotic infection in human.

## Data Availability

Data generated or analysed during this study are included in this published article. Sequences are available via NCBI GenBank. Further supporting data if required are available from the corresponding author on reasonable request.

## Abbreviations

CHRPE: Committee for Human Research, Publications and Ethics
CI: Confidence interval
CO: Cut off
ELISA: Enzyme-Linked Immunosorbent Assay
GTR + G + I: General time reversible model with a gamma distribution and proportion of invariable sites
HEV: Hepatitis E virus
HRP: Horse radish peroxidase
IgG: Immunoglobulin G
IQR: Interquartile range
KATH: Komfo Anokye Teaching Hospital
KCCR: Kumasi Centre for Collaborative Research into Tropical Medicine
KNUST: Kwame Nkrumah University of Science and Technology
MAFFT: Multiple Alignment using Fast Fourier Transform
Nc: Mean absorbance value for three negative controls
OR: Odds ratio
PCR: Polymerase Chain Reaction
RdRp: RNA-dependent RNA polymerase
RNA: Ribonucleic Acid
RT: Reverse Transcriptase
USA: United States of America
